# Superhydrophilicity and antibacterial property of a Cu-dotted oxide coating surface

**DOI:** 10.1186/1476-0711-9-25

**Published:** 2010-09-16

**Authors:** Yining Nie, Carol Kalapos, Xueyuan Nie, Monica Murphy, Riyad Hussein, Jing Zhang

**Affiliations:** 1 Tecnie Corporation, Windsor, Ontario, Canada; 2 Ontario Agency for Health Protection and Promotion, Regional Public Health Laboratory, Windsor, Ontario, Canada; 3 Department of Mechanical, Automotive & Materials Engineering, University of Windsor, Windsor, Ontario, Canada

## Abstract

**Background:**

Aluminum-made settings are widely used in healthcare, schools, public facilities and transit systems. Frequently-touched surfaces of those settings are likely to harbour bacteria and be a potential source of infection. One method to utilize the effectiveness of copper (Cu) in eliminating pathogens for these surfaces would be to coat the aluminum (Al) items with a Cu coating. However, such a combination of Cu and Al metals is susceptible to galvanic corrosion because of their different electrochemical potentials.

**Methods:**

In this work, a new approach was proposed in which electrolytic plasma oxidation (EPO) of Al was used to form an oxide surface layer followed by electroplating of Cu metal on the top of the oxide layer. The oxide was designed to function as a corrosion protective and biocompatible layer, and the Cu in the form of dots was utilized as an antibacterial material. The antibacterial property enhanced by superhydrophilicity of the Cu-dotted oxide coating was evaluated.

**Results:**

A superhydrophilic surface was successfully prepared using electrolytic plasma oxidation of aluminum (Al) followed by electroplating of copper (Cu) in a Cu-dotted form. Both Cu plate and Cu-dotted oxide surfaces had excellent antimicrobial activities against *E. coli *ATCC 25922, methicillin-resistant *Staphylococcus aureus *(MRSA) ATCC 43300 and vancomycin-resistant *Enterococcus faecium *(VRE) ATCC 51299. However, its Cu-dotted surface morphology allowed the Cu-dotted oxide surface to be more antibacterial than the smooth Cu plate surface. The enhanced antibacterial property was attributed to the superhydrophilic behaviour of the Cu-dotted oxide surface that allowed the bacteria to have a more effective killing contact with Cu due to spreading of the bacterial suspension media.

**Conclusion:**

The superhydrophilic Cu-dotted oxide coating surface provided an effective method of controlling bacterial growth and survival on contact surfaces and thus reduces the risk of infection and spread of bacteria-related diseases particularly in moist or wet environments.

## Background

Hospital infections caused by all kinds of bacteria sicken millions of people. For instance, methicillin-resistant *Staphylococcus aureus *(MRSA) alone infects about 880,000 patients and accounts for 8% of all hospital infections in the U.S.A [1]. MRSA infections continue to cause concerns not only in the healthcare setting but also in farm animals [2]. Outbreaks have occurred in schools, professional and high school sports facilities, military training centers and prisons. Frequently-touched surfaces that are likely to harbour bacteria and be a potential source of infection such as clinic settings, doorknobs, push plates, handles and taps are often made of stainless steels and aluminium (Al) alloys partially due to their appearance or low cost. However, laboratory tests show that MRSA, *Escherichia coli *(*E. coli*) and other bacteria can survive on stainless steel and aluminium surfaces for a long period of time, whereas exceptionally high levels of those pathogen bacteria are eliminated within 90 minutes on copper (Cu) surfaces [3,4]. The use of antimicrobial copper materials as environmental surfaces has the potential to reduce bacterial levels in clinical settings and beyond, which would subsequently reduce the amount of bacteria available for transmission between humans through the contact of common surfaces [2].

Cu is an essential trace element for humans and most other forms of life, including bacteria. In humans, Cu is taken up by ingestion of food and water, but not significantly by touching Cu surfaces. One of the key functions of the skin is to be a barrier against outside agents. In contrast to the human skin, the bacterial envelope can be penetrated by Cu. In case of excess, Cu ions can cause a cascade of negative events in bacterial cells. Possible mechanisms of the antibacterial actions of Cu include oxidative damage by reactive oxygen species (ROS), loss of cell envelope integrity and leakage of small solutes, and interference with other essential elements, such as Zn and Fe [4-6]. Interference with integrity of the cell envelope may be part of copper's effect on bacteria. While gram-positive bacteria (e.g., *Staphylococcus aureus*) may maintain cell envelope integrity, gram-negative bacteria (e.g., *E. coli*) lose cell envelope integrity [2,5].

Cu surfaces can inactivate microbes including *E. coli*, MRSA, and *C. difficile *bacteria [3-7]. The contact killing of bacteria by Cu is rapid and complete. On a dry Cu surface, bacteria perish in a matter of minutes [2,5] and do not have time to develop biofilms, different from aqueous systems [5]. Some heterotrophic organisms have been found to have Cu tolerance in aqueous environments [5,8]. The high antibacterial efficacy displayed by Cu and Cu alloys, at typical indoor temperature and humidity levels, favours the use of Cu-made settings as antimicrobial materials in indoor environments where humidity is low [2]. However, the settings in public transit, public recreation and sport facilities and bathrooms are usually contaminated with moisture and water. Water is an essential factor for bacterial growth and biofilm formation. The characteristics of an organism and its surrounding environment are among the important factors that may affect the survival of bacterial cells on surfaces [9].

Due to its low cost and light weight, Al-made settings are widely used in the healthcare, schools, public facilities and transit systems. To utilize the effectiveness of Cu in eliminating pathogens for those Al items, a method would be to coat the Al items with a Cu coating. However, such a combination of Cu and Al metals is susceptible to galvanic corrosion because of their different electrochemical potentials. In this work, a new approach was proposed, in which electrolytic plasma oxidation (EPO) of Al [10-12] was used to form an oxide surface layer followed by electroplating of Cu metal on the top of the oxide layer. The oxide was designed to function as a corrosion protective and biocompatible layer [13-15], and the Cu in the form of dots was utilized as an antibacterial material. The antibacterial property enhanced by superhydrophilicity of the Cu-dotted oxide coating was evaluated.

## Methods

### Materials and preparation of Cu/oxide coatings

Square coupons (25 mm × 25 mm × 5 mm) were cut from commercially-available pure copper (C11000) and pure aluminum (AA1100) plates. The coupons were ground and polished with SiC abrasive papers to obtain a surface roughness of ~0.1 μm. For preparation of EPO coatings, the Al coupons (as the anode) and a stainless steel electrode (as the cathode) were connected to a pulsed DC power supply operating at a 2000 Hz. An alkali-silicate solution (~8 g/l Na_2_SiO_3_) was used as an electrolyte with addition of KOH to adjust the pH value to pH 12-13. During the coating process, the current density was maintained at ~0.15 A/cm^2 ^and the voltage increased gradually with process time. A 5 minute treatment time was selected to obtain an oxide surface layer on Al coupons with the layer thickness of approximately 5-8 μm at the deposition rate of 1.5 μm min^-1 ^[10,14]. For preparation of Cu dots on the top of the oxide coatings, a DC power supply was used to electroplate Cu metals on the EPO-treated Al coupons for 20-25 minutes. The electroplating solution was 20 g/l CuSO_4 _and 200 g/l sodium citrate in distilled water. The current density was setup at ~0.008 A-cm^-2^. The coverage of Cu dots on the oxide layer surfaces increased with treatment time.

### Observation of surface hydrophilicity and coating morphology

~0.3 ml red wines were dropped onto surfaces of the Cu and Al metals as well as the coated coupon to investigate their hydrophilic properties. The spreading behaviors of the liquid were observed. Scanning Electron Microscopy (SEM, JEOL JSPM-5200) with energy dispersive X-ray analysis (EDX) was used to examine the as-prepared Cu-dotted oxide coating at a 15 kV voltage operating condition. Prior to the SEM observation, the coating was sputter-coated with platinum to minimize surface charging. The SEM (operated at a 10 kV acceleration voltage) was also used to observe a relatively safe, nonpathogenic *E. coli *JM101 strain tested on the Cu-dotted oxide coating. 0.05 ml of the *E. coli *suspension media at a 10^8 ^colony-forming unit per milliliter (CFU/ml) level (JM101 strain 85 V 0103 from Ward's Natural Sci.), used as a model system to study effect of coating surface morphology on bacterium inactivation, was placed onto the surface of a sterilized Cu-dotted oxide coating coupon and naturally dried at room temperature for 12 hours. The coupon was not coated with platinum before loaded in the SEM, thus the EDX spectrum would show carbon content more clearly when there is a bacterium.

### Tests of antibacterial activity

The tested samples were the Cu-dotted oxide coatings on Al coupons as well as Cu coupon surfaces and Al coupon surfaces for comparison, which were designated with sample names as S3, S1, and S2, respectively. The bacteria tested included *E. coli *ATCC 25922, MRSA ATCC 43300, and *E. faecium *ATCC 51299 (vancomycin-resistant *Enterococcus*). The technique used for determination of the effect of different surfaces on the viability of pathogenic bacteria was described as follows.

The coupons were placed into vials of 95% ethanol until used. The coupons were removed from the ethanol and quickly passed through a Bunsen Burner flame to accelerate drying before put into sterile plastic Petri dishes. Those coupons were then exposed to UV light for 6 minutes. A few bottles of pre-prepared SPC (standard plate count) agar were melted using a microwave, allowed to cool slightly and placed in a 46°C water bath.

15 Petri dishes were arranged into 5 rows of 3 Petri dishes. The first row remained empty and was served as the "control" surface. The second to fourth rows of the 3 dishes were to hold the three kinds of the testing coupons, in which the dishes were respectively numbered with testing hours of 1, 2 and 3 hrs. The fifth row named "SMA" (sterile melted agar) plates was extra control plates with each pouring set. These plates served to ensure that the SPC agar used was sterile and that the pouring of the plate did not result in contamination of the SPC bottle. The side of the coupon exposed to the UV light remained in the "up" position.

A 0.5 MCF (MacFarland) suspension was made of the bacteria being tested. A 0.5 MCF suspension was equivalent to 1.5 × 10^8 ^CFU/ml. 0.1% Peptone water was used for all dilutions and resulted in a volume of approximately 30-40 ml with a concentration of 75 to100 CFU/0.1 ml (i.e., 750 to 1000 CFU/ml). Then, 0.4 ml aliquots of the suspension were added to the surface of each of the coupons and to each of the 3 Petri dishes labeled "C". The inoculum was spread out slightly with the intention to make the coverage on all the coupons and in the "control" Petri dishes alike. The bacterial suspension covered about half of the metallic coupon surfaces (S1 and S2). However, the S3 coupons caused the suspension media to spread over their entire upper surfaces.

A set of 15 new Petri dishes was labeled, corresponding to the plates that contained the coupons. These dishes were used to hold the 0.1 ml aliquot that was removed from the "control" plates and from the surfaces of the coupons at 1, 2 and 3 hours, respectively. SPC agar was then added to those plates and mixed with the aliquot. Once the agar was hardened the plates were incubated at 35°C for 48 hours. After 48 hours the colonies in each of the plates were counted and recorded. All of the tests above were repeated two times (T1 and T2). The average results of the repetitive tests were used for the comparison analysis. The antibacterial properties can be assessed by bacterial reduction (*BR*):

(1) $$BR=\frac{N_{s}-N_{c}}{N_{c}}\times 100\%$$

where *N_c _*and *N_s _*were the average numbers of bacteria on the "control" surfaces and on the tested coupons, respectively.

## Results

### Hydrophilic behavior of the coupons

**Figure 1:**
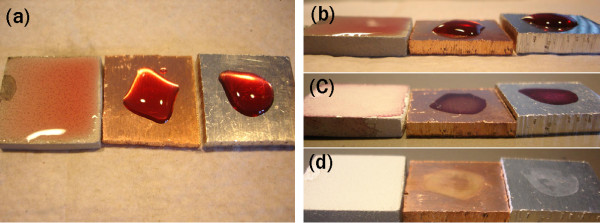
**Red wines on test coupons: Cu (middle), Al (right) and Cu/oxide coating (left)**. (a, b) drops of red wines at the beginning, (c) after 12 hours, and (d) after being run through water.

For a Cu-dotted oxide coating, the contact angle is too small to be precisely measured directly by a contact angle gonoiometer because water spreads on the coating surface. However, the contact angle is believed less than 5°. Thus, the Cu-dotted oxide surface can be considered as superhydrophilic. In order to observe the difference in hydrophilicity of coupons S1 (Cu plate), S2 (Al plate) and S3 (Cu-dotted oxide coating), ~ 0.3 ml colorful red wines were placed on the coupon surfaces as shown in Figure 1(a). While the wines only covered one fourth to one third of the Cu and Al coupon surfaces, the wine spread and covered almost all the top surface of the coated coupon, indicating that the coating surface was superhydrophilic. These phenomena were also true for water and the bacteria suspension media. The superhydrophilic property would cause the liquid layer much thinner on the top of the coating surface than on the Cu and Al coupon surfaces. The liquid layers on the Cu and Al plates had a thickness of 2-3 mm (Figure 1(b)). The thickness of the liquid layer on the coating surface, however, was less than 1 mm. All the surfaces were still wet within the period of 3 hours' testing at the tested condition. After 12 hours in the ambient air, the wines totally dried (Figure 1(c)). The dried wines on the coupon surfaces could be readily flushed away with tap water. While the coating surface had no obvious residual stains after the washing, the stains could be seen on the Cu and Al surfaces (Figure 1(d)). The stain on the Al surface could be removed by mopping the surface, whereas the stain mark on the Cu plate could not be mopped away.

It should be noted that the oxide layer without Cu can also have a superhydrophilic surface if the oxide layer has a large thickness (and thus a rough surface). However, the oxide layer prepared in this work was relatively thin and did not have superhydrophilicity. The contact angle of water on the thin oxide coating surface was in a range of 40-60°. The liquid drops can be spread only 10-15% wider on the oxide-only layer than on the Cu and Al surfaces. The superhydrophilic property of the coating was resulted from the combined surface morphology of the oxide layer and Cu dots, as shown in the following SEM observation.

### SEM/EDX analysis

**Figure 2:**
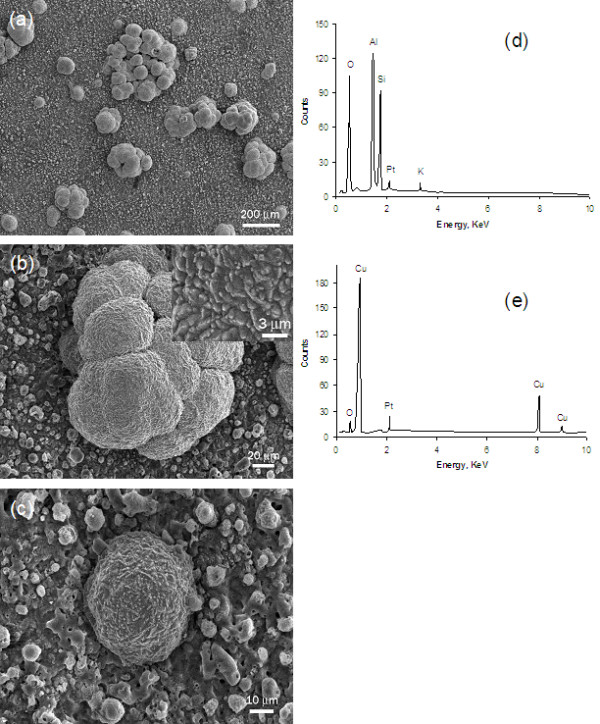
**SEM micrographs and EDX analysis of the Cu-dotted oxide coating**. (a) Cu-dotted oxide coating, (b) cauliflower-like Cu dots, (c) single Cu dot, (d) EDX obtained from the oxide layer, and (e) EDX obtained from a Cu dot.

SEM micrographs and EDX analysis results were presented in Figure 2, which showed that the coating comprised an oxide layer and Cu dots. The dots on the top of oxide layer formed either individual spherical particle with 30-70 μm in diameter or cauliflower-like clusters around 200 μm in size, Figure 2(a). Micron-sized features (bumps) on the dotted surfaces were revealed in the inset on Figure 2(b). During the electroplating process, the Cu dots initially formed as individuals (Figure 2(c)). Their sizes grew and cauliflower-like clusters were also produced when the electroplating process time increased. The EDX spectrum showed that the oxide layer contained Al-Si-O elements (Figure 2(d)). The oxide prepared using the EPO process was known to have a combination of α-Al_2_O_3_, γ-Al_2_O_3 _and mullite phase structures [10,14]. The dots were almost pure Cu with minimum oxygen (Figure 2(e)). The Pt in the spectra of Figure 2(d, e) was due to the sputter coating to make the coupon surface conductive prior to the SEM observation. The coverage of the Cu dots was estimated to be 15-20%. Although it is difficult to precisely determine the total surface area of micron-sized spherical and cauliflower-like Cu dots, their total surface area should be larger than the surface area of the Cu "flat" plate.

The hybrid Cu-dotted oxide coating also exhibited a very good adhesion, which was evidenced by the Scotch tape tearing experiments. The tape experiments did not tear any Cu dots and oxide off the coated coupon surface. The oxide layer was metallurgically grown from the base material during the EPO process. The oxide had a relatively rough surface morphology which provided mechanical anchor sites for Cu dots to easily adhere to. The electroplating of Cu dots should be initiated at the "weak" points where the plating current could leak through the oxide layer. Increasing the deposition treatment time would make the Cu dots grow larger, form more cauliflower-like clusters and cover a greater surface area of the oxide layer. The coating shown in the SEM micrograph (Figure 2(a)) was treated by the EPO for 4 minutes followed by electroplating of Cu for about 20 minutes.

**Figure 3:**
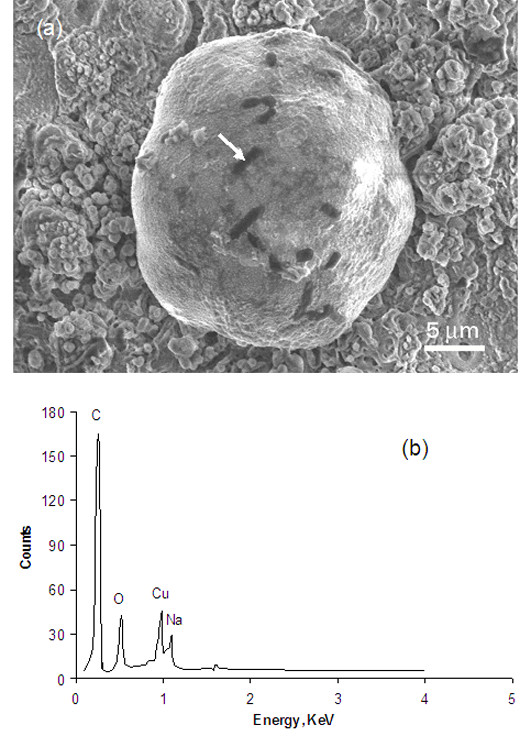
**SEM micrograph and EDX analysis of the Cu-dotted oxide coating**. (a) A Cu dot with bacteria and (b) EDX obtained from the dark area (as indicated by the arrow in (a)).

The SEM micrograph, Figure 3(a), shows the test results of the nonpathogenic *E. coli *(JM 101 strain 85 v133) culture which was placed on a Cu-dotted oxide coating and dried in the air at ambient temperature for 12 hours. The EDX analysis on the dark areas showed very high carbon content compared to the surrounding areas (Figure 3(b)). The dimension of each dark area matched the shape (i.e., rod-shaped) and size (typically 2 μm long and 0.5 μm in diameter) of an *E. coli*. The cell volume in *E. coli *can increase in certain circumstances [16]. Therefore, the dark areas were believed to be the dehydrated *E. coli *bacteria which experienced 12 hours drying in the air, osmotic shocking in the vacuum, and carbonizing under exposure to SEM electron beams. The *E. coli*, having a shape of rod, had a tendency to stick onto Cu dot surfaces since it was much easier to find the bacterium on the Cu dots than on the oxide surface. The Cu-dotted surfaces became smoother after the *E. coli *culture test (Figure 3(a)) than before the test (Figure 2(c)). The bacterial suspension contained NaCl and phosphate, which may have caused preferential dissolution of the bumps on the Cu dot surfaces during the tests, resulting in a chemical "polishing" effect.

### Antibacterial Property

The antibacterial test results of Cu coupons (S1), Al coupons (S2), and Cu-dotted oxide coatings (S3), together with control plates (C) and SMA plates (SMA), were summarized in Table 1. The numbers of bacteria at 0 hour were the amount of bacteria initially placed on the tested coupons and control plates. The bacteria population on the control samples (labeled with "C" in the table) slightly decreased (for *E. coli*), increased (for MRSA), or decreased initially and then increased (for *E. faecium*) during the testing hours. The numbers of bacteria on the control samples (*N_c_*) at 1 hr, 2 hrs and 3 hrs were used as references for evaluation of the bacterial reduction rate (i.e., *BR *in Equation 1) of the coupons tested at the corresponding hours. The larger the difference of the bacterial counts between the control samples (*N_c_*) and coupon samples (*N_s_*), the better the antibacterial performance of the coupons.

**Table 1:** CFU counts on Cu plates (S1), Al plates (S2) and Cu-dotted oxide coatings (S3) and their BRs.

	C		S1		S2		S3		SMA		BR, %		
**Time (hrs)**	**T1**	**T2**	**T1**	**T2**	**T1**	**T2**	**T1**	**T2**	**T1**	**T2**	**S1**	**S2**	**S3**

E. coli ATCC 25922													
0	46	104	-	-	-	-	-	-	-	-	0	0	0
1	42	108	22	69	44	99	9	24	0	0	-42	-2	-60
2	38	71	4	15	47	62	0	24	0	0	-84	6	-52
3	37	79	0	6	43	68	3	1	0	0	-96	1	-98
MRSA ATCC 43300													
0	145	93	-	-	-	-	-	-	-	-	0	0	0
1	167	103	111	67	143	76	1	46	0	0	-34	-20	-64
2	176	114	147	73	152	50	4	6	0	0	-26	-35	-96
3	193	131	90	34	162	59	16	0	0	0	-64	-36	-100
E. faecium ATCC 51299													
0	153	161	-	-	-	-	-	-	-	-	0	0	0
1	126	138	136	130	166	120	96	91	0	0	1	9	-31
2	143	148	96	93	154	81	53	70	0	0	-35	-19	-52
3	163	193	80	75	148	101	3	1	0	0	-56	-28	-99

Table 1 clearly shows that the Cu plate (S1) could inactivate the *E. Coli *ATCC 25922 effectively, particularly after 2 hours tests, while the Al plate (S2) could not inhibit the bacteria. The Cu-dotted oxide coating could kill most of the bacteria and functioned even better than the Cu plate, especially in the first hour. The excellent inactivation performance of the Cu-dotted oxide coating was also true for MRSA ATCC 43300 bacteria while the Cu plate could only eliminate about one quarter to one third of the MRSA bacteria in the first and second test hour. A significant amount of the MRSA still survived after the 3 hours, likely due to a thick layer (i.e., 2-3 mm) of the suspension liquid which reduced the probability of the bacteria contacting the Cu killing surface. Interestingly, however, the Al plate also had somehow inhibiting effects on the MRSA bacteria. For the tests against *E. faecium *ATCC 51299, the Cu and Al plates did not show any antibacterial function until the second test hour. While nearly half of the bacteria remained after 3 hours, the Cu-dotted oxide coating almost completely eliminated the *E. faecium *at the third hour. The *E. faecium *seemed more copper-resistant than the *E. coli *and MRSA.

**Figure 4:**
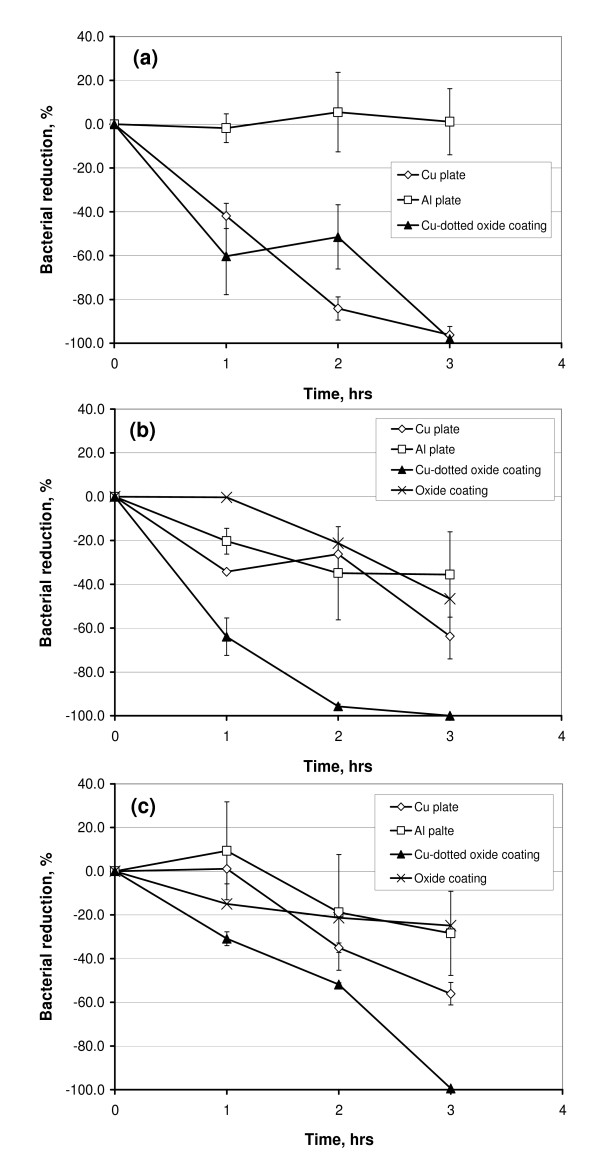
**Bacterial reduction (*BR*) vs. time of the Cu plate, Al plate and Cu-dotted oxide coating tested with (a) *E. coli *ATCC 25922, (b) MRSA ATCC 43300 and (c) *E. faecium *ATCC 51299**. The oxide coatings without Cu were also included in (b) and (c).

Figure 4 illustrates the differences in bacterial reductions (*BR*) between Cu plates, Al plates and Cu-dotted oxide coatings against different bacteria at the 1st, 2nd and 3rd hours. Seen from the test data at 1 hour, the Cu-dotted oxide coating could inactivate bacteria much quicker than the Cu plate in the tested conditions. The coating could eliminate more than 96% of all kinds of the bacteria within 3 hours. Although the antibacterial activity of the Cu plate against the *E. coli *was very effective after the 3 hours test, the plate could only inactivate around 60% of the MRSA and *E. faecium *after the same testing periods. The large difference in the first-hour test results between the Cu plate and the Cu-dotted oxide coating was believed to be due to their surface morphologies. The Cu-dotted oxide coating is superhydrophilic (Figure 1(b)), which allows the bacterial suspension to spread on the coating surface and thus better contact the Cu dots. However, the interaction of the organisms with the metallic copper plate in this setup was rather indirect, since the Cu plate surfaces were covered with a thick layer (2-3 mm in thickness) of the bacterial suspension liquid during the 3-hour tests. It should be noted that the antibacterial property of the oxide coatings against MRSA and *E. faecium *were also presented in the Figs. 4(b) and 4(c), showing that the oxide had no significant antibacterial activity.

## Discussion

*E. coli *is a gram negative, rod-shaped bacterium. *E. coli *can survive for brief periods outside the body [16-19]. MRSA is often sub-categorized as community-acquired MRSA (CA-MRSA) or health care-associated MRSA (HA-MRSA). HA-MRSA affects people in healthcare settings. New CA-MRSA strains have become the most common cause of cultured skin infections among individuals seeking emergency medical care. These strains also commonly cause skin infections in athletes, prisoners and soldiers [17]. *Vancomycin resistant Enterococcus faecium *(VRE) is a well-known contaminant strain in indoor environments [18,19]. Like MRSA, VRE is considered a super-bug and can be highly drug-resistant. VRE is an important concern not only because a VRE infection is difficult to treat in clinical practice but also because VRE clones can spread within a hospital as well as between entire countries.

*E. coli*, MRSA and VRE are facultative anaerobic organisms, i.e., they do not require oxygen for metabolism but can survive in oxygen-rich environments [16,18]. The ability of the copper to prevent those microbial environmental contaminations has been reported in reference [20], which demonstrates that reduction in viable count of MRSA is greater than that of *E. faecium *after 3 hours exposure to copper, while reduction of both MRSA and *E. faecium *is less than that of *E. coli*. The same trend was found in this study for the Cu plate coupon (Figure 4). However, the antibacterial activity to all the three microorganisms was greatly enhanced by the Cu dots deposited on the oxide surface. Such a Cu-dotted surface had benefits in two aspects: a superhydrophilicity, which could spread the suspension liquid and allow a greater contact surface area, and a micron-scaled texture, on which bacteria intended to attach firmly (Figure 3(a)).

Under the testing conditions, a desiccant stress effect from a more "drying" surface due to the roughness and the superhydrophilicity of the Cu-dotted oxide coating should not play an important role in underlying antibacterial mechanisms. Otherwise, a difference in *BR *between *E. coli *and MRSA would have appeared in Figure 4 for the Cu-dotted oxide coating, since the MRSA is among the most desiccation-resistant microorganisms. MRSA can survive in severe water deficit while gram-negative bacteria (e.g., *E. coli*) are more susceptible to the air-drying effect [9]. In fact, during the 3-hour testing periods, the coating surface was still visibly wet, and 0.1 ml aliquot could be readily obtained and removed from each coupon surface for the antibacterial performance tests.

If the bacterial suspension was spread all over the Cu plate using a cover glass, the Cu plate would have an equal or even better antibacterial performance as the Cu-dotted surface. However, this paper intended to study the antibacterial property of their natural surfaces without external interruption. The test results showing the enhancement in antibacterial activity were due to the surface morphology of the Cu-dotted oxide coating. The superhydrophilicity of the coating is attributed to its non-patterned surface. If a surface has a regularly textured pattern, superhydrophobicity is usually apparent [21,22]. However, this was not the case for this study. The coating surface exhibited superhydrophilicity which resulted from its irregular surface morphology. Therefore, incorporating a Cu-dotted oxide coating offers the potential for a method of controlling bacterial growth and survival on contact surfaces and thus reduces the risk of infection and spread of bacteria-related diseases. This would be particularly important in environments where moisture or wet conditions are involved, cleaning practices are done occasionally or ineffectively due to inaccessibility, or nature of cleaning products used are not completely effective in removing and killing bacteria.

## Conclusion

This work has evaluated the effectiveness of a superhydrophilic Cu-dotted oxide coating surface in the inactivation of *E. coli *ATCC 25922, MRSA ATCC 43300 and *Enterococcus faecium *ATCC 51299. The superhydrophilicity of the Cu-dotted oxide coating on the aluminum substrate was due to combination of the surface morphologies of the oxide layer and the spherical and cauliflower-like Cu dots. The antibacterial laboratory results showed that both the Cu plate and Cu-dotted surface greatly reduced microorganisms whereas Al did not exhibit a significant effect in the inhibition of bacteria. However, the Cu-dotted oxide surface performed even better than the Cu plate surface, particularly in the early stages of contact with the bacterial suspension cultures. The enhanced antibacterial properties resulted from the superhydrophilic behavior of the Cu-dotted surface that allowed bacteria to contact Cu more effectively due to spreading of the bacterial suspension media.

## Competing interests

The authors declare that they have no competing interests.

## Authors' contributions

All authors have contributed to and approved the final manuscript. YN performed the experimental data analysis and wrote and edited the manuscript. CK performed the microbiology laboratory tests. XN conceived, designed and supervised the study. MM managed the lab facilities and supervised the antimicrobial tests. RH prepared the coatings. JZ advised in study design and in writing the manuscript
